# Modified Roll Flap Soft-Tissue Augmentation at Single-Stage Implant Placement: A Digital-Scan–Verified Case Report

**DOI:** 10.3390/dj13100483

**Published:** 2025-10-21

**Authors:** Kamen Kotsilkov, Hristina Maynalovska, Zdravka Pashova-Tasseva

**Affiliations:** Department of Periodontology, Faculty of Dental Medicine, Medical University of Sofia, 1431 Sofia, Bulgaria; k.kotsilkov@fdm.mu-sofia.bg (K.K.); z.pashova@fdm.mu-sofia.bg (Z.P.-T.)

**Keywords:** dental implant, roll flap, soft tissue augmentation, soft tissue dimension maintenance

## Abstract

**Background and Objectives:** Adequate peri-implant soft tissue dimensions are essential for health, hygiene, and esthetics. When ridge volume is sufficient, phenotype modification may avoid bone grafting. This case report describes a pedicled roll flap performed concurrently with single-stage implant placement after spontaneous socket healing, without bone substitute, and assesses soft-tissue stability with serial intraoral scans. **Clinical case:** A single-tooth edentulous site underwent prosthetically driven, fully guided implant placement. A modified roll flap with vertical and palatal incisions was prepared; the de-epithelialized crestal connective tissue was elevated and rolled into a buccal envelope to augment thickness. No graft material was used. A provisional crown conditioned the emergence profile. Follow-up included photographs, radiographs, and intraoral scan superimpositions at 2 weeks, 3–4 months, 8 months, and 14 months after implant treatment. Healing was uneventful. Buccal soft-tissue thickness increased, keratinized mucosa was preserved, and midfacial levels remained stable. Emergence profile and papillae integrated harmoniously. Crestal bone levels were stable radiographically. Digital scans corroborated soft-tissue thickness maintenance. No donor-site morbidity occurred. **Conclusions:** In healed sockets with adequate bone, a modified pedicled roll flap at implant placement can thicken the peri-implant phenotype and achieve stable esthetic integration without bone substitutes.

## 1. Introduction

Modern implant therapy offers reliable solutions for restoring function and esthetics in both partially and completely edentulous patients. Long-term studies confirm that implant-supported rehabilitations can achieve predictable success beyond 10 years, though mechanical and biological complications still threaten their survival [1,2,3].

Successful implant placement requires not only adequate bone volume but also favourable peri-implant tissue conditions. Both hard and soft tissues are essential for predictable outcomes at placement and long term [4]. In particular, sufficient soft tissue thickness and keratinized gingiva support peri-implant health, facilitate hygiene, and ensure harmonious esthetics.

The literature data further emphasize the close interrelationship between buccal bone morphology and the stability of the overlying facial soft tissues. Several clinical and experimental studies investigating immediate implantation, with or without guided bone regeneration (GBR), have shown that intact buccal bone height is strongly associated with the preservation of facial soft tissue contours. In contrast, buccal bone deficiency frequently results in gingival recession, which negatively affects both esthetics and long-term peri-implant stability [5,6].

In this context, soft tissue augmentation procedures have gained increasing clinical relevance. By enhancing tissue volume and improving mucosal conditions, these techniques contribute to the long-term stability of peri-implant health and esthetics. The following case report illustrates a clinical approach to soft tissue augmentation in the esthetic zone, highlighting its role in achieving predictable functional and esthetic outcomes.

The concept of the periodontal phenotype encompasses both the gingival phenotype, which includes gingival thickness and the width of keratinized gingiva, and the bone morphotype, referring to the buccal bone plate [7]. Common types of alveolar bone deficiencies include intra-alveolar defects, dehiscences, fenestrations, and horizontal or vertical bone defects [8,9].

According to the literature, several factors increase the risk of peri-implant mucosal recession, including implant malposition, buccal bone plate deficiency, insufficient soft tissue thickness, lack of keratinized tissue, the periodontal attachment status of adjacent teeth, and surgical trauma. Marginal bone loss of less than 1.5 mm is generally accepted as a reference threshold for successful implant treatment one year after functional loading [10,11,12].

Deficiencies in the periodontal phenotype can compromise implant survival. In cases with a thin phenotype, pre-implant hard and soft tissue augmentation may improve functionality and reduce the risk of gingival or bony dehiscence [13,14]. Soft tissue thickness has been shown to be critical for peri-implant stability. Linkevicius et al. [14] reported that patients with thin peri-implant soft tissues experienced a mean marginal bone loss of 1.5 mm within one year compared to 0.3 mm in patients with thick gingiva. Similar findings from other studies confirm that soft tissue thickness is a key determinant of bone stability [15,16], and improving the tissue quality contributes to maintaining stable peri-implant bone levels [17].

In addition to soft tissue thickness, the clinical parameters of keratinized mucosal width and mucosal margin mobility are also important considerations. However, longitudinal studies have not consistently confirmed an association between the keratinized mucosal width and changes in marginal bone levels following implant therapy [1]. From a clinical perspective, soft tissue augmentation (STA) procedures serve two primary objectives: (i) to increase the width of attached and keratinized peri-implant mucosa, and (ii) to enhance the overall soft tissue thickness. Achieving these goals provides both short- and long-term biological, functional, and esthetic benefits [18].

Beyond their role in optimizing soft tissues, STA procedures also contribute to bone preservation by improving the adaptation of peri-implant soft tissues. A systematic review conducted by Thoma et al. summarized that augmentation of the attached keratinized mucosa leads to reduced bleeding scores and improved peri-implant bone levels. Moreover, increasing mucosal thickness through autogenous grafting has been shown to minimize crestal bone loss, further emphasizing the relevance of soft tissue augmentation in implant therapy [19].

Augmentation of keratinized mucosa has been shown to improve clinical parameters such as probing depth (PD) and bleeding on probing (BOP). Procedures aimed at increasing soft tissue volume, such as the use of connective tissue grafts, demonstrate additional benefits in esthetic outcomes. Furthermore, when soft tissue augmentation (STA) is combined with bone augmentation procedures, the incidence of marginal soft tissue recession is reduced compared to bone augmentation alone. Free gingival grafting has been reported to enhance peri-implant health by improving bleeding indices and reducing inflammation of the peri-implant mucosa. However, evidence regarding its influence on marginal bone loss remains inconsistent. While some studies failed to establish a significant relationship between STA with free gingival grafts and marginal bone preservation [20,21], others have documented reduced marginal bone loss in grafted areas compared with non-augmented sites [22].

Similarly, procedures designed to augment the volume of peri-implant soft tissues have produced variable outcomes. Certain studies found no significant association between soft tissue augmentation and marginal bone loss, whereas others reported stable marginal bone levels following augmentation with connective tissue grafts [16,17]. In clinical practice, soft tissue augmentation has become an essential adjunct in cases with a thin periodontal phenotype, buccal bone deficiency, or esthetic demands. By increasing keratinized mucosa and enhancing tissue dimension, such procedures not only support peri-implant health but also contribute to stable esthetic outcomes. Although evidence on their influence on marginal bone levels is mixed, clinical experience consistently shows that optimizing soft tissue conditions improves predictability and reduces the risk of biological or esthetic complications. For these reasons, soft tissue augmentation was chosen in the present case to enhance peri-implant stability and ensure long-term functional and esthetic success [16,17,23]. Although soft-tissue augmentation is well established, there is limited evidence on modifications of the pedicled roll flap performed simultaneously with the single-stage implant placement in spontaneously healed sockets. The present case introduces a novel modification of this technique, demonstrating its potential to enhance peri-implant soft-tissue thickness without the use of bone substitutes.

## 2. Materials and Methods—Clinical Case

**Figure 1:**
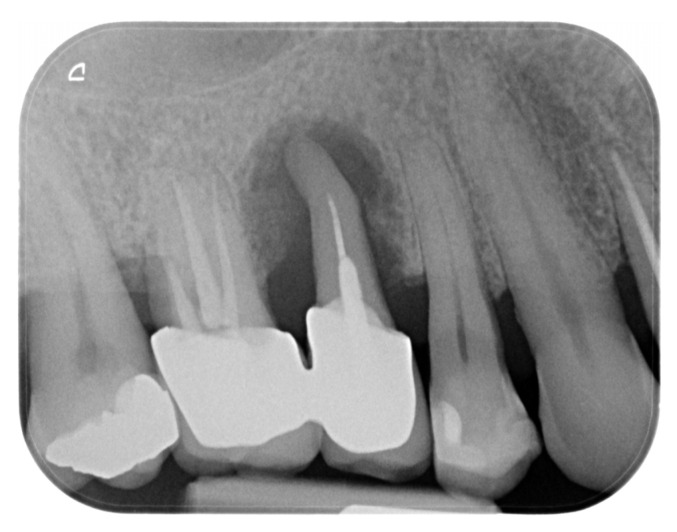
Initial X-ray status prior to clinical examination.

A 45-year-old male patient, non-smoker, non-diabetic, and without systemic comorbidities, was referred for periodontal treatment. Perioperative radiographic assessment identified a diffuse peri-radicular radiolucency at the maxillary right second premolar, consistent with an endo-periodontal lesion and confirming the need for extraction (Figure 1).

Following an atraumatic tooth removal, the clinical examination revealed the morphology of the residual socket, which exhibited intact alveolar walls but a thin buccal plate, highlighting the importance of careful post-extraction management with blood cloth protection with a collagen sponge sutured to the socket walls and sealed with a tissue glue.

**Figure 2:**
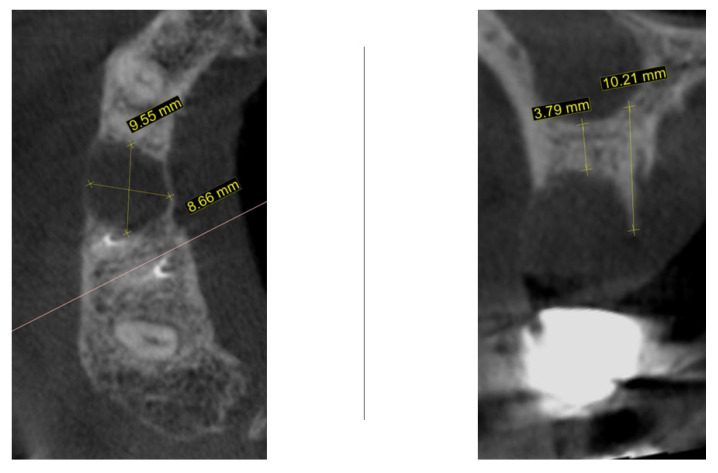
CBCT one month following atraumatic extraction.

One month later, cone-beam computed tomography (CBCT) was performed to assess the morphology of the post-extraction site (Figure 2). For the region of interest (second right maxillary premolar), digital impressions were obtained using iTero Element Flex (Align Technology, Inc., Tempe, AZ, USA) and processed with Exoplan software, version 3.1 Rijeka (Exocad GmbH, Darmstadt, Germany). The iTero scanner applies a primarily surface-based registration complemented by anatomical landmarks, ensuring accurate fit and alignment. Using confocal imaging, it acquires detailed surface topography through point-and-stitch reconstruction, producing high-precision 3D models of teeth and soft tissues without the need for powder. Digital scans obtained during planning and at 3, 4, and 8 months post-surgery were superimposed using Exoplan (Exocad GmbH) software. Two-dimensional changes were assessed: (1) horizontal dimension—from the platform of the planned implant position to the corresponding buccal mucosal point, and (2) vertical dimension—from the implant platform to the buccal mucosal margin of the planned or placed restoration. The cross-sectional view demonstrated a large radiolucent zone with width of 9.5 mm and bucco-lingual dimension of 8.6 mm. In the sagittal plane, the residual vertical bone height measured 3.7 mm with a preserved palatal bony wall (10.2 mm). Although the assessment of these CBCT findings usually necessitates a bone augmentation procedure before implant placement, an alternative decision was taken to wait until the natural healing process is complete.

**Figure 3:**
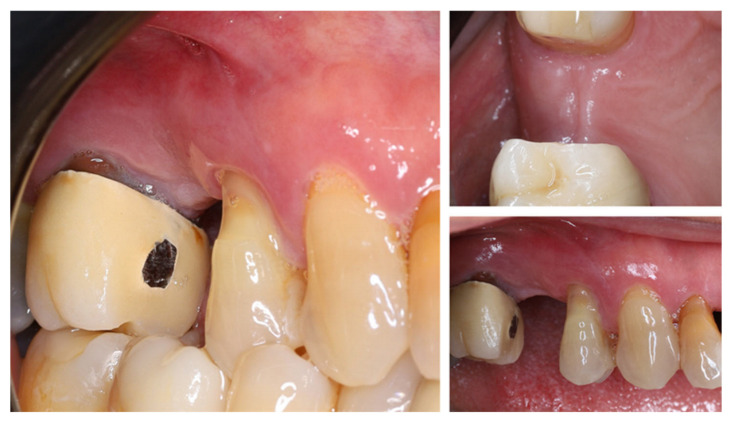
Clinical status of the patient after healing—vestibular and occlusal view.

One year after tooth extraction, the clinical examination revealed complete soft tissue healing (Figure 3). At the buccal aspect of the alveolar ridge, the usual post-extraction horizontal ridge defect was observed. Post-extraction alveolar ridge collapse is a common clinical condition after tooth extraction. Horizontal and vertical bone loss can reach up to 60% within 2 years after tooth extraction, with most occurring within the first year after extraction.

This volume loss is most pronounced in the bucco-lingual direction and occurs more frequently in the absence of adequate buccal wall thickness of the dental alveolus (<1 mm) [24].

**Figure 4:**
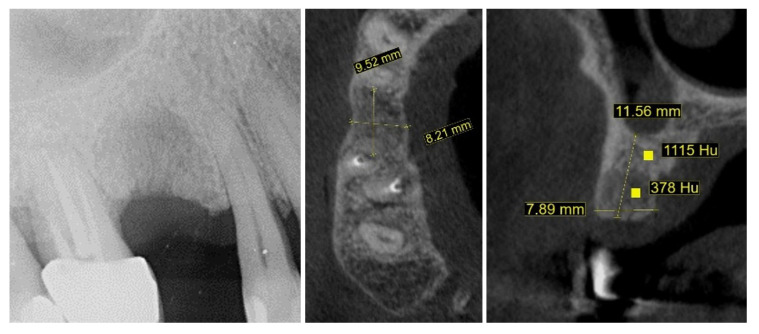
One year after autogenous healing of the extraction site.

New cone-beam computed tomography (CBCT), performed to assess the morphology of the healed ridge, demonstrated a complete healed crestal bone with a bucco-lingual width of 8.2 mm. In the sagittal plane, the total vertical bone height measured 11.5 mm, suggesting adequate dimensions for implant placement without additional bone augmentation. The bone density measurement presented a high BMD value in the apical part of the crest while the density in the newly formed bone was relatively low (Figure 4).

**Figure 5:**
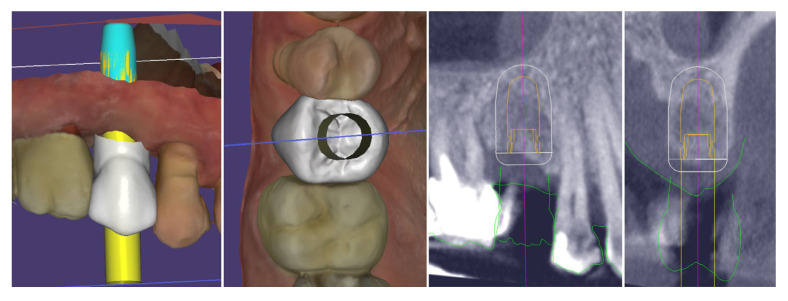
Digital planning of the dental implant.

Digital planning of the dental implant was performed. It was determined that there was sufficient crestal bone volume to place the implant in the most appropriate prosthetically oriented position, for which a surgical guide was prepared for fully guided implantation (Figure 5).

**Figure 6:**
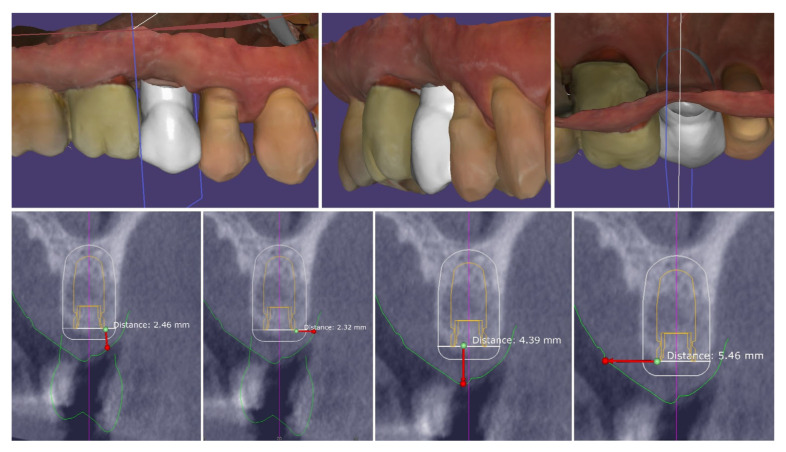
Soft tissue dimensions analysis.

The performed measurements showed adequate soft tissue dimension (≥3.5 mm) for stable peri-implant tissues crestally, 4.3 mm, and palatally, 5.4 mm, but at the buccal site, a pronounced volume deficiency was detected (2.3 mm soft tissue thickness), for which a soft tissue augmentation procedure was planned (Figure 6).

The analysis of the crestal edentulous area revealed adequate soft tissue thickness to perform the modified roll flap for implant placement:

- Bucco-lingual width of the crestal keratinized mucosa ≥ 4 mm;
- Mesio-distal width of the crestal mucosa (to the adjacent teeth)—∅ of the implant + min 3 mm;
- Height of the buccal keratinized mucosa ≥ 1 mm;
- Height of the palatal keratinized mucosa ≥ 3 mm;
- Thickness of the crestal mucosa ≥ 2 mm.

**Figure 7:**
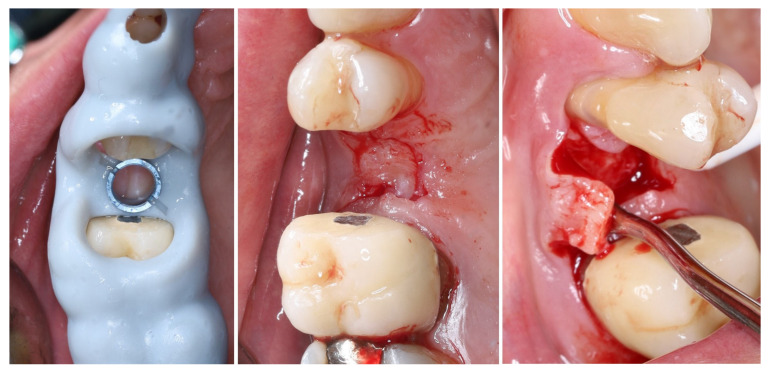
Flap design and flap reflection.

Intraoperatively, the flap preparation included (Figure 7) crestal flap preparation by marking the borders of the implantation area through the surgical guide with the tissue punch followed by crestal U-shaped incision with the blade (15C) perpendicular to crestal mucosa.

The preservation of unprepared areas of the crestal soft tissue to the adjacent teeth, transformed them into the future peri-implant papillae that can be used to ensure stable primary flap closure, protecting the peri-implant bone from post-surgical resorption.

The U-shaped incision was extended mesially and distally with oblique incisions at the border of the crestal roll flap, separating the peri-implant papillae into anatomic (unprepared part) and surgical parts. The crestal part of the flap was de-epithelized with a 15C blade and microsurgical scissors and was elevated to full thickness with a periodontal chisel (36/37 Rhodes Back-Action chisel, Hu-Friedy, Chicago, IL, USA).

The buccal flap is prepared in partial thickness with the two incisions known from mucogingival surgery, described by De Sanctis and Zucchelli, a split-thickness incision, with the blade parallel to the bone, keeping the periosteum intact, and a split-thickness incision parallel to the external mucosal surface, releasing muscle insertions from the flap. A critical aspect of the procedure was the preservation of the periosteal layer on the buccal wall of the residual socket. This minimally invasive flap design, characterized by split-thickness preparation and the absence of vertical releasing incisions, ensured sufficient vascularization of the flap and minimized the risk of scar formation on the buccal surface [25,26].

**Figure 8:**
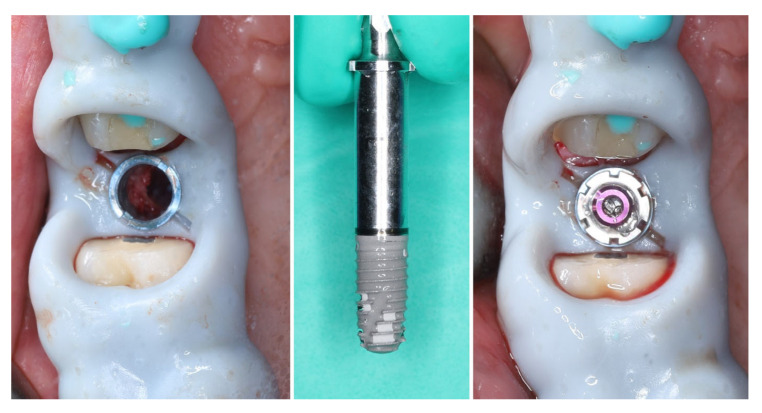
Guided implantation protocol.

The implant was placed using a digitally designed surgical guide to ensure prosthetically driven positioning. Intraoperative images demonstrate the sequence of guided placement: preparation of the osteotomy through the guide with visualization of the prepared site (left), dental implant (T3Pro, ZimVie Inc., Palm Beach Gardens, FL, USA) before placement (middle), and final implant insertion (right) with insertion torque ~50N/cm. The use of a surgical template allowed precise three-dimensional implant placement, optimal angulation, and preservation of surrounding soft and hard tissues (Figure 8).

**Figure 9:**
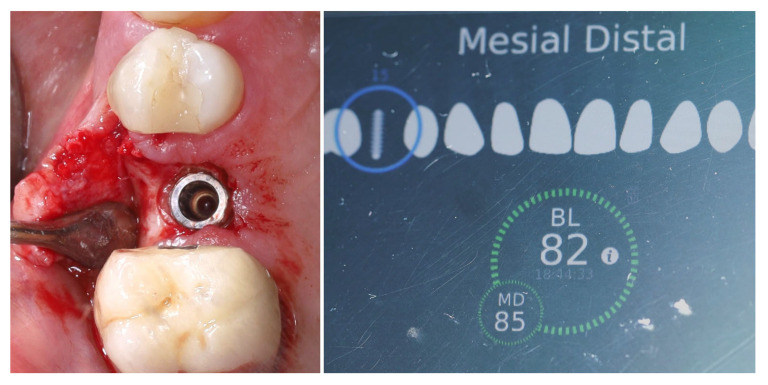
Implant placement and initial stability.

The selected implant was positioned in the prosthetically driven position, with appropriate bone thickness at the buccal aspect and surrounded by healthy soft tissues (left). Another advantage of this surgical approach is the possibility to adapt the implant insertion depth corresponding to the soft tissue thickness in order to provide enough soft tissue thickness to enhance the stability of the peri-implant bone. The intraoperative measurements confirmed excellent primary stability, with ISQ values of 82 bucco-lingually and 85 mesio-distally (right). These parameters indicated optimal mechanical anchorage of the fixture within the alveolar bone, ensuring favourable conditions for osseointegration (Figure 9).

**Figure 10:**
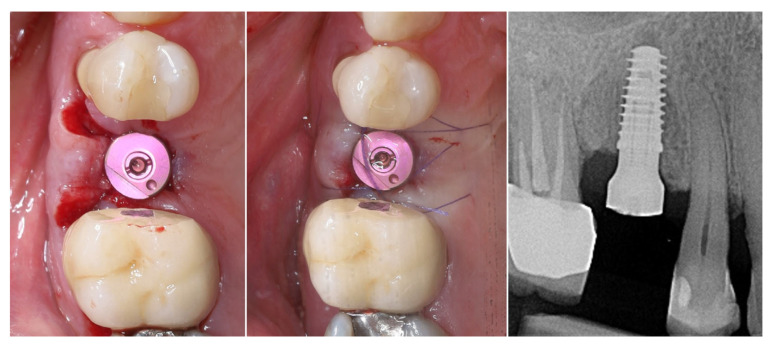
Clinical and X-ray view after implant placement and roll flap adaptation.

A multi-purpose healing abutment, serving also as a scan body (encode abutment ZimVie Inc., Palm Beach Gardens, FL, USA), was connected, providing proper concave emergence profile in the critical contour zone of the transmucosal area. The de-epithelized crestal flap was rolled and fixated to the inner surface of the buccal flap with a horizontal mattress suture (7/0 PGA). The buccal flap was fixed to the “anatomical” part of the peri-implant papillae (6/0 PGA). The buccal positioning of the rolled connective tissue creates a thickened gingival margin, intended to improve peri-implant soft tissue stability and long-term esthetic outcomes. The surgical site shows adequate adaptation of the flap margins around the healing abutment, enhancing the primary healing, thus reflecting proper flap management and atraumatic technique. The 2D X-ray performed after implant placement shows a proper implant position corresponding to the morphology of the alveolar crest (Figure 10).

**Figure 11:**
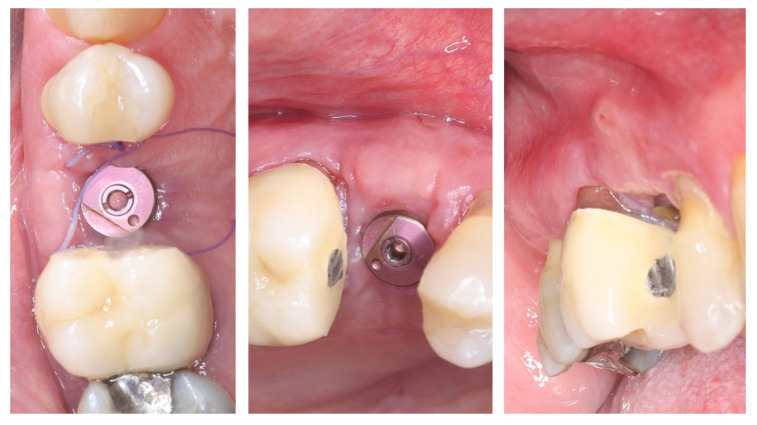
Clinical views before and after suture removal (14 days post-op).

Figure 11 illustrates the peri-implant soft tissue condition 14 days after simultaneous implant placement and roll flap augmentation at suture removal. The buccal flap shows uneventful healing with adequate tissue thickness and favourable contouring around the healing abutment. The mucosal margin appears stable, with absence of dehiscence or inflammation, indicating successful integration of the rolled flap. Different views demonstrate proper adaptation of the peri-implant mucosa, confirming the effectiveness of the roll flap in enhancing buccal soft tissue thickness and achieving a harmonious gingival architecture.

**Figure 12:**
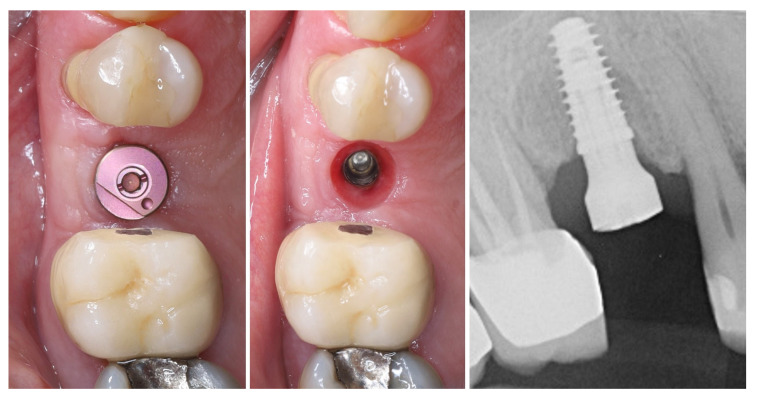
Mature peri-implant mucosa and stable crestal bone after roll flap augmentation (3 months post-op).

Three months postoperatively, the peri-implant site demonstrates complete mucosal healing with stable, well-contoured soft tissues. The healing abutment is visible in situ (left), with healthy, keratinized mucosa surrounding the transmucosal interface. Following removal of the healing abutment (middle), the implant platform shows a mature and stable soft tissue collar without signs of inflammation, recession, or dehiscence. The X-ray control reveals stable crestal bone levels without signs of bone remodelling (right) (Figure 12). The roll flap augmentation resulted in increased buccal tissue thickness and harmonious gingival architecture, providing favourable conditions for prosthetic restoration.

**Figure 13:**
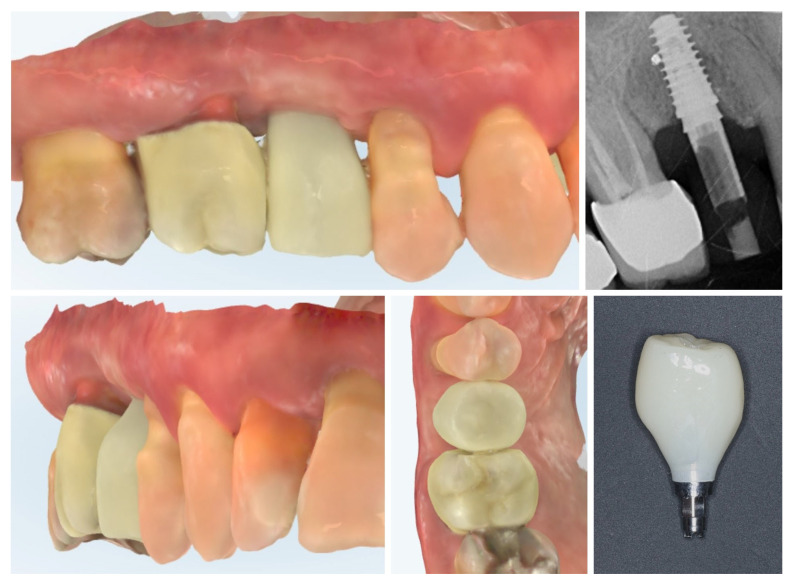
Digital impressions and provisional crown fabrication (3 months post-op).

The clinical workflow progressed to digital impression of the multi-purpose healing abutment and provisional crown fabrication. The emergence profile of the Ti-base and the transmucosal part of the crown follows the profile of the healing abutment exactly, thus avoiding any changes in the critical contour zone of the transmucosal area in order to avoid any crestal bone remodelling after placement of the provisional crown. Intraoral scans (upper and lower rows) confirm the stable peri-implant mucosal margin achieved through roll flap augmentation, providing favourable conditions for an esthetic emergence profile (Figure 13).

**Figure 14:**
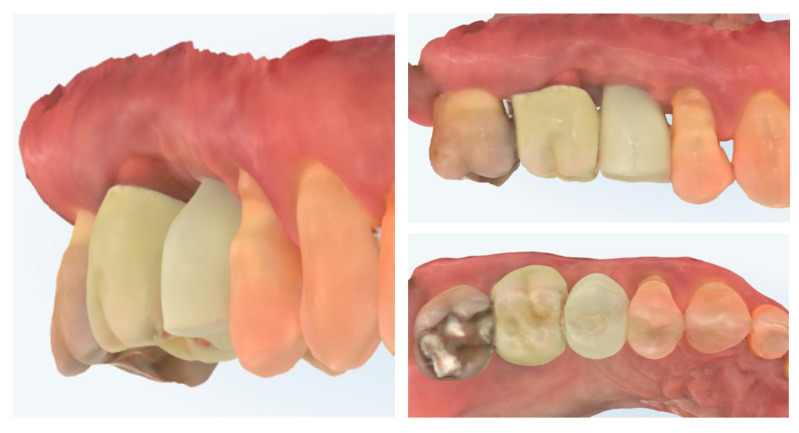
Intraoral scan 1 month after provisional crown placement (4 months post-op).

One month after provisionalization, intraoral scans demonstrate stable peri-implant soft tissue contours around the temporary crown. The mucosal margin exhibits favourable adaptation and maturation, with preservation of the buccal volume achieved through roll flap augmentation. The temporary restoration supports the peri-implant mucosa, guiding the emergence profile for the definitive prosthesis (Figure 14.).

**Figure 15:**
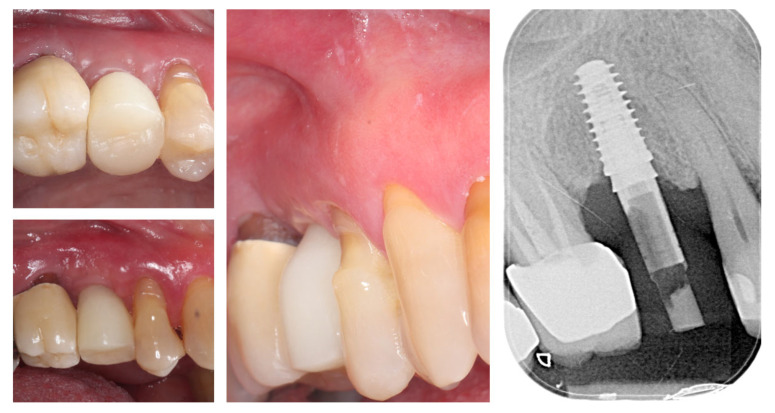
Clinical and radiographic evaluation at 4 months with provisional crown (7 months post-op).

At 4 months after provisional crown placement, clinical examination reveals stable peri-implant soft tissues with a healthy mucosal margin and increased buccal and interdental soft tissue thickness around the temporary crown. The soft tissue integration demonstrates maturation and stability, with no signs of inflammation, recession, or dehiscence. Radiographic evaluation (right) confirms proper implant positioning with intimate bone-to-implant contact and preservation of crestal bone levels, indicating successful osseointegration and tissue stability at this stage of provisionalization (Figure 15.).

**Figure 16:**
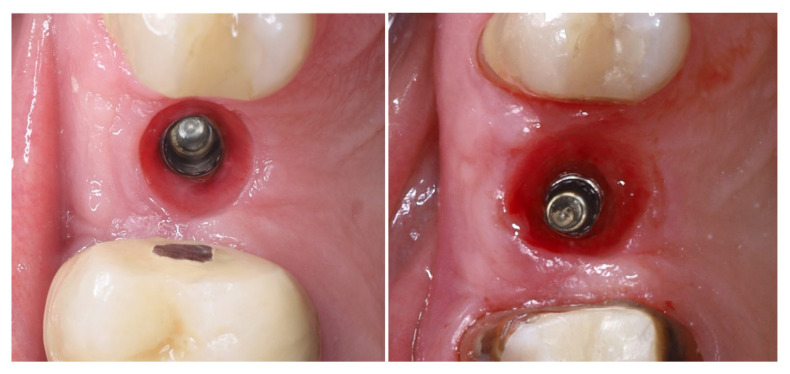
Peri-implant mucosal contours prior to definitive restoration (8 months post-op).

Clinical views of the implant site prior to final prosthetic restoration show a well-formed peri-implant mucosal collar with healthy soft tissue contours. The buccal mucosa demonstrates adequate thickness, consistent with the roll flap augmentation performed at the time of implant placement. The soft tissue margin appears stable without signs of inflammation, ensuring favourable conditions for the subsequent impression and definitive crown placement (left). The comparison with the situation before placement of the provisional crown (right) shows the increase in the soft tissue volume induced by the emergence profile of the crown (Figure 16).

**Figure 17:**
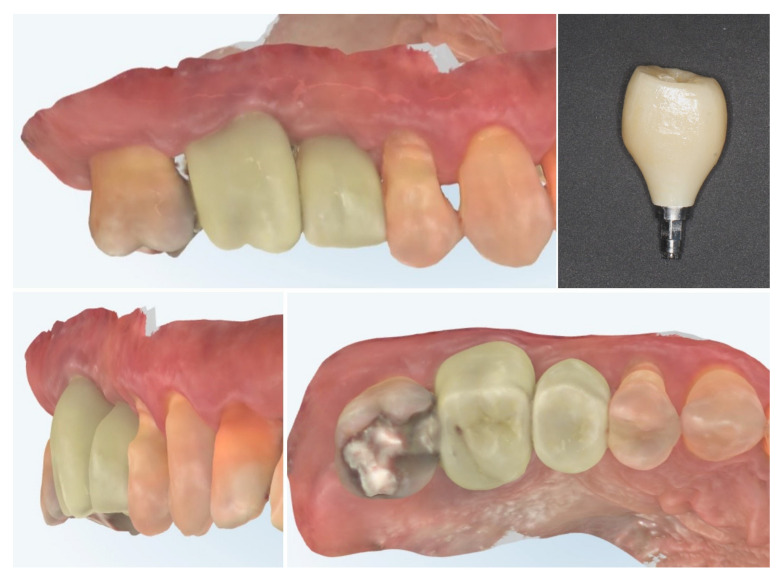
Clinical view of the final implant-supported and tooth-supported crowns (8 months post-op).

Digital scans obtained at delivery of the definitive prosthetic reconstructions show stable integration of both the implant-supported crown in region 15 and the full-contour zirconia crown on tooth 16 (Figure 17). The emergence profile of the implant-supported crown completely copies the profile of the temporary crown in order to not induce changes in the crestal bone. The peri-implant soft tissues demonstrate healthy contours and harmonious adaptation to the restoration’s emergence profile. The occlusal and proximal relationships are well-preserved, ensuring functional loading and esthetic integration with adjacent teeth. No soft tissue recession or volumetric loss is evident, confirming the stability of the roll flap augmentation and the favourable outcome of the prosthetic phase.

**Figure 18:**
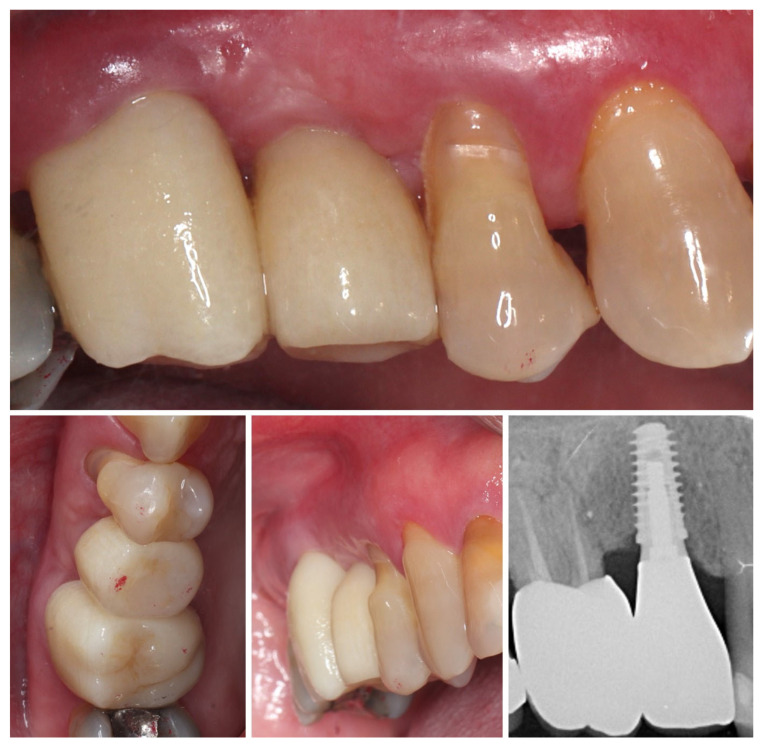
Clinical appearance and X-ray control 6 months after definitive prosthetic treatment (14 months post-op).

Clinical view at 6 months following delivery of the definitive prosthetic restorations demonstrates stable peri-implant and periodontal soft tissues (Figure 18). The mucosal margin is healthy, with adequate buccal thickness and harmonious integration of the implant-supported crown in region 15 and the adjacent tooth-supported crown in region 16. No signs of inflammation, mucosal recession, or soft tissue collapse are evident, confirming long-term stability of the roll flap augmentation and successful functional and esthetic rehabilitation. The X-ray control shows stable crestal bone levels.

**Figure 19:**
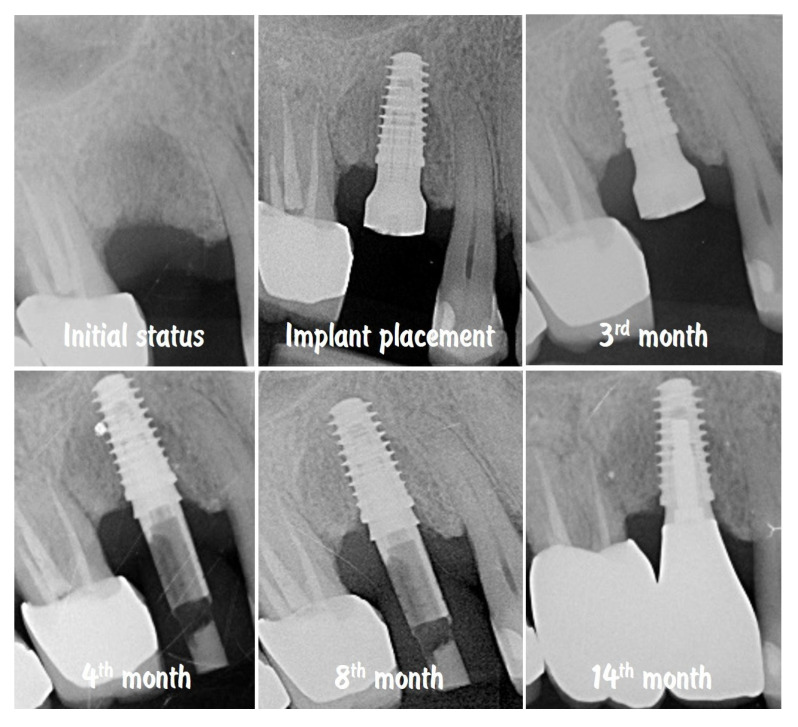
Stability of the crestal peri-implant bone.

The comparison of the X-rays shown in Figure 19 presents the stability of the crestal bone levels prior to implant placement to the 14th month after implantation. The combination from minimally invasive implant site preparation, provision of an adequate peri-implant soft tissue thickness, and proper emergence profile of the implant-supported restoration ensured the protection of the bone volume without signs of remodelling or resorption.

**Figure 20:**
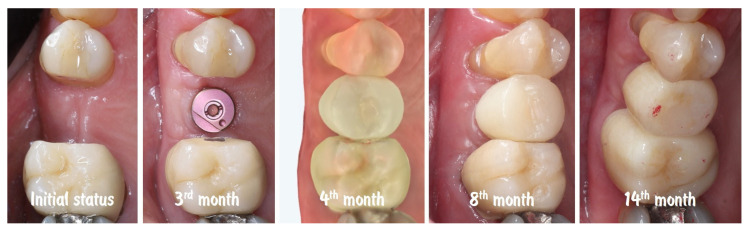
Dynamic of soft tissue maturation and development.

Figure 20 shows the dynamic of soft tissue maturation and development. The combination from soft tissue augmentation procedure with the modified roll flap and soft tissue conditioning with the temporary crown led to complete resolution of the horizontal defect of the alveolar crest and provided adequate soft tissue thickness for long-term stability of the implant-supported restoration.

**Figure 21:**
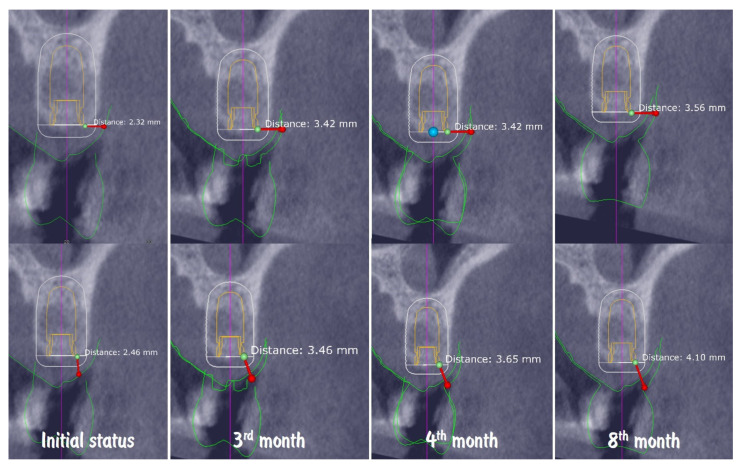
Digital volumetric analysis of the increase in the peri-implant soft tissues.

Digital volumetric analysis illustrates the stable increase in the peri-implant soft tissues over time, presented in (Figure 21). The buccal soft tissue dimensions increased from 2.3 mm horizontal thickness and 2.4 mm vertical thickness at baseline (Day 0) to 3.4 mm horizontal thickness and 3.4 mm vertical thickness at the 3rd month, showing approximately 1 mm thickness increase from the soft tissue augmentation procedure. One month after the connection of the temporary crown, the vertical thickness increased to 3.6 mm. At the time of the final crown placement, the horizontal thickness was 3.5 mm, and the vertical thickness was 4.10 mm, demonstrating the potential of the emergence profile of the crown to induce increase in the soft tissue thickness. The soft tissue thickness increase for the period of 8 months was 1.24 mm (53.45%) in horizontal dimension and 1.64 mm (66.67%) in vertical dimension.

**Figure 22:**
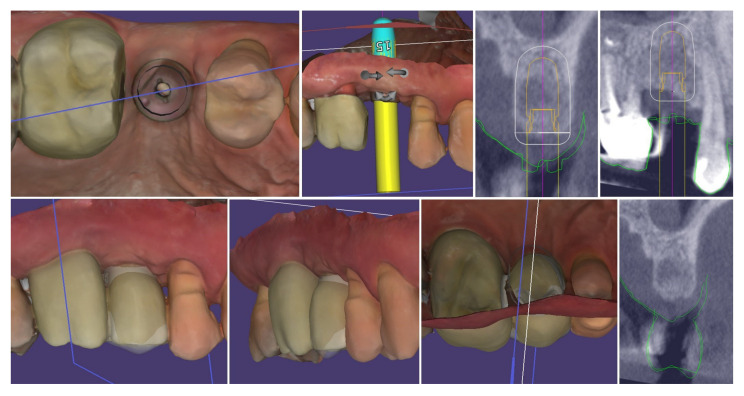
Congruence of the implant position with digital planning and of the prosthetic planning with the final crown.

Figure 22 demonstrates the capabilities of the modern dental technology to assist the clinician in achieving accurate and reproducible results. The top row presents the congruence of the implant position with digital planning, while on the bottom row, the similarity between the prosthetic planning and the actual final crown is presented.

Table 1 summarizes horizontal and vertical soft-tissue thickness at baseline and during subsequent follow-up intervals (2 weeks, 3 months with provisional crown, 4 months post-provisional, 8 months with definitive crown, and 14 months post-definitive). Clinical and radiographic findings confirm uneventful healing, increased buccal thickness, stable mucosal margins, and preservation of crestal bone levels, supporting long-term soft-tissue stability.

A limitation of the present clinical case is the relatively short observation period. Functional and esthetic outcomes were documented up to 6 months after delivery of the definitive prosthesis and 14 months after implant placement. Extended follow-up is necessary to confirm whether the favourable peri-implant tissue conditions and soft tissue augmentation achieved with this technique can be maintained over time.

## 3. Discussion

A frequent limitation at the intended implant sites is an insufficient band of keratinized mucosa (supracrestal attached tissues). Adequate keratinized mucosa facilitates plaque control, is associated with reduced early marginal bone loss [27,28], and improves peri-implant esthetics around the suprastructure [29]. The faster progression of bone destruction in peri-implantitis compared with periodontitis further underscores the importance of sufficient peri-implant soft-tissue volume [29,30]. Concurrently, rising patient esthetic expectations place additional emphasis on gingival augmentation to achieve a natural-looking outcome in the implant area [31]. The objectives of soft-tissue augmentation around implants are twofold: to re-establish an adequate width of attached mucosa and to increase tissue thickness. When mucosal thickness is <3 mm, augmentation can be performed at various stages—before implant therapy, during implant placement, at second stage uncovering, or after prosthetic loading [32], with earlier soft-tissue management generally favoured in the literature.

The literature consistently underscores the need to establish a sufficient quality of peri-implant soft tissue with appropriate clinical parameters. These objectives are most predictably achieved with soft-tissue augmentation procedures, most commonly using autogenous connective tissue grafts [33]. Autogenous grafts are broadly classified by histologic composition into free gingival grafts (FGG) and connective tissue grafts (CTG). FGG is primarily employed to increase the width of keratinized mucosa, often in conjunction with an apically displaced flap, whereas CTG is indicated to increase peri-implant tissue thickness. When the graft is not completely detached from the donor site and retains its vascular pedicle, it is considered a pedicled graft; this category includes techniques based on roll flaps and related variants [23,34,35]. In contrast to gingival recession coverage, where a thin gingival phenotype and the presence of apical keratinized tissue guide the indication for CTG, the management of peri-implant soft-tissue dehiscence typically necessitates a graft regardless of the existing keratinized mucosa. Such augmentation can be performed in conjunction with immediate implantation [29] or at the time of implant exposure during second-stage surgery [36,37,38]. In our case, employing a pedicled roll flap at implant placement effectively increased soft-tissue thickness and keratinized mucosa, supporting stable peri-implant contours and favourable esthetics at follow-up [39,40].

Two principal approaches are used to harvest connective tissue grafts (CTG). The first involves raising a partial-thickness flap and separating the underlying connective tissue—with or without periosteum—yielding a subepithelial CTG. The second approach harvests a free gingival graft (FGG) that is then de-epithelialized extra-orally. The subepithelial approach allows for the primary closure of the donor site, favouring primary intention healing and reduced postoperative sensitivity [41]. However, it carries a risk of necrosis of the primary palatal flap if overly thinned and may produce a graft with lower collagen density because part of the superficial lamina propria remains with the flap [42].

With extra-oral de-epithelialization, the autogenous graft is essentially an FGG converted to CTG. Its main advantage is access to the dense, collagen-rich lamina propria located in the superficial palatal mucosa; deeper (more apical) layers contain adipose and glandular tissue that may impede early revascularization and nutrient diffusion [43,44]. This technique is typically fast to harvest, but the donor site heals by secondary intention, which can increase postoperative sensitivity and bleeding in some patients [45]. Irrespective of technique, several palatal anatomical safeguards apply: the a. palatina major typically courses at a mean distance of ~12 mm from the CEJ (verify your abbreviation “CEG” → CEJ), and a ≥2 mm safety margin from the coronal incision to the CEJ helps prevent palatal recession. In patients with a flat palatal vault, the artery may lie as close as ~7 mm from the CEJ [46].

Roll flaps are a form of pedicled soft-tissue augmentation in which the partially dissected connective tissue remains attached to the donor site, thereby preserving vascular supply, reducing volumetric contraction, and enabling a minimally invasive protocol [47]. First described by Abrams (1980) and since refined through multiple modifications [48,49], roll-type procedures can increase the keratinized tissue width at implant or pontic sites, achieve favourable esthetics, and limit morbidity by avoiding a second donor wound while maintaining superior graft vascularization [50]. Subsequently, the technique underwent a number of modifications and was adapted to the needs of dental implantology [47].

Surgically, the primary flap is prepared with two vertical incisions joined by a paracrestal horizontal incision in partial thickness (length adapted to the clinical defect). The connective tissue segment is liberated on three sides, de-epithelialized, and rolled beneath the buccal flap. Variants fall broadly into roll techniques and the vascularized interproximal periosteal connective tissue flap (VIP-CTG). In classic roll methods, the pedicled flap remains attached to the vestibular flap and is rotated under it; indications include small peri-implant soft-tissue defects at various stages of implant therapy and the contouring of pontic sites for fixed prostheses [51,52,53]. The advantages of these techniques are that they allow an increase in the horizontal volume of the alveolar ridge buccally and to resemble its contour corresponding to the roots of the adjacent teeth. A major disadvantage of some of these approaches, which significantly limits their use, is the mobilization of the entire volume of the crestal mucosa, which exposes the peri-implant crestal bone and leads to secondary healing, which inevitably follows a larger volume of bone remodelling and loss of the horizontal bone level. In other variations, only the crestal volume of tissue corresponding to the diameter of the implant being placed is used, which is de-epithelialized and placed in a subepithelial envelope buccally, which ensures primary healing and does not involve the horizontal bone level, but is applicable only in situations with fully healed bone after extraction (Type 4 implantation) with sufficient bucco-lingual width for implantation without bone augmentation [51,53,54,55,56,57]. The current consensus also emphasizes the need to tailor such soft tissue augmentation strategies to the initial phenotype, keratinized mucosa, and long-term stability requirements [58].

One modification presented in this case report is the “Modified Roll flap”, which significantly expands the indications for the application of this technique applying the principles of mucogingival surgery [23]. This flap design, as opposed to traditional roll flap design, has several advantages, allowing access to the buccal surface of the bone crest, providing the opportunity for bone augmentation, if necessary, which could be combined with additional gingival grafts if more pronounced soft tissue augmentation is needed, and provides the possibility for coronal flap advancement and stable flap fixation. This flap design preserves the periosteum of the buccal bone wall, which reduces the volume of postsurgical bone loss, while allowing for de-epithelialization of the anatomical papillae and coronal displacement of the buccal flap, which not only allows for a horizontal tissue volume increase in the edentulous area but also for vertical gingival augmentation and root coverage of the teeth adjacent to the edentulous area [59].

In our case, this U-shaped, pedicled roll flap performed concurrently with implant placement facilitated the uneventful healing and buccal contour thickening observed at 3–4 months and maintained at the 14th month after implant placement, supporting a stable emergence profile and pleasing esthetics. The crestal bone remained stable without any sign of remodelling or resorption.

For the vascularized interpositional periosteal connective tissue graft (VIP-CTG), this second group comprises a pedicled flap that remains attached to the palatal tissues mesially or distally, according to the defect location. VIP-CTG is indicated for larger peri-implant soft-tissue deficiencies and can be combined to simultaneously augment soft and hard tissues. It may be applied at different stages of implant therapy (e.g., immediate placement, second-stage surgery) [60] and is also useful for developing soft tissues in pontic/bridge areas [52]. A related option is the palatal-sliding flap performed with a window approach, which mobilizes the palatal mucosa vestibularly toward the deficit and, within anatomical limits, helps compensate interproximal papilla height [61]. For evidence synthesis and clinical positioning, the reviewed literature supports the need for adjunctive soft-tissue augmentation to achieve stable peri-implant mucosal conditions. Multiple techniques can effectively increase soft-tissue volume, yet the procedure with the highest predictability remains debated. Overall, implants managed with soft-tissue augmentation (STA) tend to show higher long-term survival and a lower risk of peri-implant mucositis/implantitis [62]. In our case, the defect was small-to-moderate, so a pedicled roll flap was preferred over VIP-CTG to minimize morbidity while preserving vascularity. This choice yielded uneventful healing, buccal phenotype thickening, and stable mucosal margins at 3–4 months and maintained at 14 months, supporting a favourable emergence profile and esthetics.

## 4. Conclusions

This case demonstrates that a pedicled roll flap performed concurrently with single-stage implant placement—after spontaneous socket healing and without any bone substitute—can effectively thicken the buccal phenotype, preserve keratinized mucosa, and support a natural, tissue-friendly emergence profile with low morbidity. Serial intraoral scan superimpositions across follow-up confirmed the stable increase in soft-tissue thickness, while clinical and radiographic assessments indicated healthy peri-implant conditions and stable crestal bone levels. By maintaining graft vascularity and avoiding a second donor site, the approach reduces surgical burden and chair time yet achieves predictable esthetic integration. Within the limitations of a single case, these findings support the roll flap as a minimally invasive, reliable option for soft-tissue augmentation when ridge volume is sufficient and hard-tissue grafting is unnecessary.

## Figures and Tables

**Table 1:** Timeline of peri-implant soft tissue changes verified by digital analysis and clinical/radiographic follow-up.

	Timepoint	Horizontal Thickness (mm)	Vertical Thickness (mm)	Clinical/Radiographic Findings
1	Baseline (Day 0)	2.3	2.4	Initial thin buccal tissue
2	2 weeks post-op			Uneventful healing, stable mucosal margin
3	3 months post-op (with provisional)	3.4	3.4	Increased thickness, stable bone and mucosa
4	4 months post-provisional	3.5	3.6	Stable peri-implant mucosa around provisional crown
5	8 months post-op (final crown)	3.5	4.1	Final crown placed, harmonious soft tissue contours, and stable crestal bone
6	14 months post-definitive			Long-term stability of soft tissue and crestal bone confirmed

## Data Availability

The original contributions presented in this study are included in the article.
